# Students from single-sex schools are more gender-salient and more anxious in mixed-gender situations: Results from high school and college samples

**DOI:** 10.1371/journal.pone.0208707

**Published:** 2018-12-07

**Authors:** Wang Ivy Wong, Sylvia Yun Shi, Zhansheng Chen

**Affiliations:** Department of Psychology, University of Hong Kong, Hong Kong SAR, China; Massachusetts College of Pharmacy and Health Sciences, UNITED STATES

## Abstract

Gender segregation exists in all walks of life. One of the most common forms of institutionalized gender segregation is perhaps single-sex schooling. Because schooling experience has important influence on students’ psychosocial development, interest in gender-segregated education has been reviving over the globe. Skeptics of single-sex schooling have suggested that such schooling may increase students’ gender salience (awareness of gender in categorizations), reduce opportunities for mixed-gender interactions, and increase mixed-gender anxiety, but little evidence has been found. It is critical to explore how single-sex schooling is associated with these psychosocial outcomes in adolescents and young adults because they are in the developmental stage when the desire and need to establish mixed-gender relationships increase. We report two systematic studies on gender salience, mixed-gender friendships, and mixed-gender anxiety on 2059 high school students and 456 college students from single-sex or coeducational schools. Even with demographic background controlled, results suggested higher gender salience in single-sex school students in the high school sample, and greater mixed-gender anxiety and fewer mixed-gender friendships in these students in both samples. These differences were not moderated by student gender and were similar in first-year versus senior college students. Moreover, mixed-gender friendships, though not gender salience, appeared to engage in a possibly bi-directional mediation relationship with mixed-gender anxiety that is consistent with a vicious cycle of escalating anxiety and lack of mixed-gender interaction among single-sex school students. These findings help fill the knowledge gap about the correlates of gender-segregated schooling and shed light on the precursors of later social and achievement differences between single-sex and coeducational school students.

## Introduction

Gender segregation exists in all walks of life and begins as early as toddlerhood [1]. The most prevalent form of institutionalized gender segregation currently is perhaps single-sex schooling, a subject of intense research and educational focus around the globe. Along with the revival of single-sex education in the United States following the 2006 reinterpretation of Title IX of the U.S. Education Amendments, researchers continue to question the alleged academic and social benefits of single-sex schooling (for reviews, see [2–4]). A similar revival is seen in mainland China, where all-boys programs are being championed in an attempt to restore masculinity and academic competence in boys [5,6]. Since schooling experience plays an important role in students’ psychosocial development (e.g., see [7] for differences in sexual orientation and dating experience between single-sex and coeducational school graduates), such trends present a pressing need for comprehensive, evidence-based evaluations of the outcomes of single-sex schooling. As the most prominent difference between single-sex and coeducational schooling is the presence or absence of other-gender peers, the questions of whether single-sex schooling experience is related to students’ attentiveness to gender (gender salience) and anxiety in mixed-gender interactions have been asked by many (e.g., [2, 8–12]). For example, does gender loom larger in single-sex school students? Are single-sex school students more anxious than coeducational school students when they interact with other-gender peers? If so, is such increased anxiety related to increased salience of gender or simply to reduced mixed-gender experience?

Addressing these questions is important because mixed-gender encounters are inevitable and forming healthy mixed-gender relationships is an important developmental task. Existing studies focus heavily on achievement-related outcomes and show that single-sex and coeducational school students differ minimally on this aspect once confounds such as socioeconomic status are accounted for [4,13]. However, they provide surprisingly little knowledge about the salience of gender, mixed-gender friendships or mixed-gender anxiety. Thus, we tested current students and graduates of single-sex schools and coeducational schools on these outcomes. The research took place in Hong Kong, where single-sex schools were common during the British colonial era in the 1900s and still represent a sizeable portion in the education system today (e.g., approximately 16% of high schools) [14,15]. Here we use “gender” to refer to students’ gender, gender salience, mixed-gender anxiety, and other gender-related concepts. The term “sex” is used only when we refer to sexual orientation and single-sex schooling to follow what is much more common in the usage of these terms.

### Gender salience and single-sex schooling

Gender salience refers to the awareness of gender as a categorizing dimension [16] and it is important because school-aged children, preschoolers, and even infants readily use gender to process incoming information [17–21]. High gender salience has consequences to individuals’ psychosocial development. For example, it leads to the development of gender-role concepts and stereotypes [16,22]. When teachers created gender-salient environments in a two-week experiment, students adopted more gender stereotypes, interacted less with other-gender peers and viewed them less positively [23].

There is much debate over whether single-sex schools (e.g., [2,4]) or coeducational schools (e.g., [24]) produce greater gender salience. According to the developmental intergroup theory, gender as an identity becomes salient (cognitively accessible and relevant) under four environmental conditions—when groups are perceptually discriminable, when people are in the minority, when groups are explicitly labeled, and when groups are implicitly used [16]. This well-validated theory is often used to predict gender salience in single-sex schools. However, because single-sex schools have features that both increase and decrease gender salience, the predictions have been mixed. Most researchers hypothesize that, owing to some conditions of single-sex schools (e.g., gender labels in school names), single-sex school students are more gender-salient [2–4]. Others (e.g., [24]), emphasizing other conditions of single-sex schools (e.g., own gender being in the majority), predict the opposite. Both sides of the debate cite research on how people perform in same- vs. mixed-gender settings as supporting evidence [2,24], but such research did not directly assess gender salience in single-sex and coeducational school students.

Some studies on single-sex schooling focused on other aspects of gender cognition and their findings are mixed. For example, compared to girls in coeducational schools, Drury, Bukowski, Velásquez, and Stella-Lopez [25] found girls in single-sex schools to feel more gender-typical and pressured to conform to gender norms, but Kessels and Hannover [26] found girls in single-sex classes to have less accessible gender-related self-knowledge (lower endorsement of feminine traits and longer reaction time responding to these traits), a finding taken to reflect lower gender salience in single-sex classes. These findings are valuable in understanding how single-sex schooling relates to gender identity and stereotyping, but may not directly translate to gender salience as defined by the developmental intergroup theory [16] because the constructs are conceptually distinct [27,28]. Nevertheless, these findings point out that empirically measuring gender salience in single-sex and coeducational school students is necessary for us to directly evaluate the debate regarding which school environment highlights gender to the greater extent.

### Mixed-gender friendships, mixed-gender anxiety and single-sex schooling

Another concern regarding gender-segregated schooling experience is the potential impact on students’ mixed-gender interactions and friendships. Some school principals cited a lack of real-world comparability as a disadvantage of single-sex schooling, worrying that single-sex school students would struggle in forming mixed-gender relationships [9]. Indeed, mixed-gender interactions serve key functions in adolescents’ and young adults’ psychosocial development that are unique from same-gender interactions [29], such as providing opportunities to learn about distinct behavioral norms of the other gender, practice interpersonal skills required to communicate effectively and comfortably with the other gender in the family and workplace, and for heterosexual individuals, acquaint potential romantic partners [2,8,10,30], as well as preparing them to establish and maintain satisfactory mixed-gender relationships [29,31]. Forming positive mixed-gender friendships benefits emotional well-being, such as by diversifying social support groups and increasing self-esteem [29,32]. The quality of mixed-gender interaction is an especially important developmental task during adolescence because the time spent and the motivation to interact with other-gender peers start to increase during this period [1,33].

Reduced exposure to mixed-gender interactions was suggested to predispose individuals to experience mixed-gender anxiety [34,35], which may lead to future avoidance of mixed-gender situations, thus disrupting the process of social skills acquisition and relationship formation, resulting in a vicious cycle [29]. Interest in mixed-gender anxiety, sometimes termed “dating anxiety” or more generally “heterosocial anxiety”, has resurged recently [36]. It was found that higher mixed-gender anxiety is related to less initiatives, satisfaction and poorer performance in mixed-gender interactions [34,37], as well as delayed first dating relationship, fewer dating and sexual experiences, and more difficulties in romantic relationships for heterosexual individuals [31,32]. Besides, mixed-gender anxiety negatively affects psychological and physical well-being, including lower self-esteem and non-assertiveness and increased depression and loneliness [38–40]. However, it is important to note that not all individuals are heterosexual and mixed-gender anxiety may affect both romantic and non-romantic situations. While mixed-gender anxiety in romantic situations (referred to as dating anxiety in this study) is more relevant to heterosexual individuals, the more general form of mixed-gender anxiety in non-romantic situations (referred to as general mixed-gender anxiety) may affect individuals of any sexual orientation. Therefore, it would be meaningful to not only study dating anxiety, but also the general mixed-gender anxiety in non-romantic situations.

There is only a dearth of research on the interpersonal outcomes of single-sex schooling and they [4,13] rarely focused on mixed-gender relationships, the type of interpersonal outcomes most likely to be affected by gender segregation. A few pieces of evidence suggest that mixed-gender relationships could be negatively affected by gender segregation (but see null result in likelihood of remaining married to the first spouse [41]). For example, two studies controlling for socioeconomic background, parental education and/or religion found that, in early to middle adulthood, single-sex school graduates reported less satisfactory marriage outcomes (e.g., less happy marriage, higher rate of divorce) than coeducational school graduates [11,42]. One study found that 10^th^ grade students from single-sex schools reported lower satisfaction with other-gender friends than students from coeducational schools [43].

While the above findings are useful, the number of studies is small and the research is limited in different ways, such as assessing only restricted aspects of interpersonal outcomes (e.g., marriage outcomes) based on single-item retrospective reports [11,41,42], and failure to control for any demographic variable of single-sex and coeducational school students [43]. While a few studies touched upon other aspects of mixed-gender relationships (e.g., heterosocial adjustment) and suggested poorer outcomes associated with single-sex schooling, they are usually unpublished (e.g., [44]), dated (e.g., [44,45]), or uncontrolled (e.g., [44]).

### Possible pathways of differences

While the majority of the studies on single-sex schooling focus on identifying and describing the differences between single-sex and coeducational school students, few have tested the pathways that lead to these differences. However, some have implicated that single-sex and coeducational school students differ in various domains such as gender stereotyping and subject preferences due to the presumably higher gender salience in single-sex school students (e.g., [2,4]), implying a mediational pathway. As the awareness of oneself being of a different gender appears to trigger an individual’s concerns about gender issues during interactions [16,46] and individual’s higher salience of a social category is correlated with anxious intergroup contact [47], gender salience may mediate between school type and mixed-gender anxiety. Another pathway that may explain school differences in mixed-gender anxiety may be mixed-gender friendships. In particular, reduced mixed-gender friendships is correlated with greater mixed-gender anxiety [34,35] and single-sex schools perceivably provide few opportunities for mixed-gender friendships, so mixed-gender friendships may mediate between school type and mixed-gender anxiety.

### This study

This study aims to address the research gap in the single-sex schooling debate by directly measuring and comparing gender salience, mixed-gender friendships and mixed-gender anxiety in single-sex and coeducational school students in two samples in Hong Kong, one at high school (i.e., 2059 current single-sex and coeducational high school students) and the other at college (i.e., 456 single-sex and coeducational school graduates currently studying in a coeducational college). We also aimed to contribute to the literature by better controlling for confounding variables. Most prior studies comparing single-sex and coeducational school students included no controls, and those that did usually only included family socioeconomic status and, for studies on academic performance, sometimes the students’ preexisting ability [3,4]. Controlling for confounds like these diminished the school differences in academic achievement [4,48]. Although these confounds may be less of an issue when the dependent variables (such as the current variables on mixed-gender anxiety and gender salience) are not closely related to factors affecting school choice (such as academic performance), we followed the advice of controlling for some potentially confounding background differences between single-sex and coeducational school students [3,4,8,48]. In Hong Kong, around 16% of high schools are single-sex and all colleges are coeducational. Single-sex classes in coeducational schools are extremely rare. The academic quality of high schools is indicated by three bands, with Band 1 being the highest and Band 3 being the lowest. As in most regions, the allocation of students into schools is not random. We controlled for parental income and education, as socioeconomic status is one of the most important control variables in single-sex schooling research [8]. We additionally controlled for school banding and the numbers of brothers and sisters to rule out potential differences due to the academic quality of schools and gender composition at home [33,49]. For the college sample, we also controlled for sexual orientation and whether the students were studying in a male-dominated, female-dominated, or gender-balanced faculty.

Finally, we explored whether the potential school difference in mixed-gender anxiety was mediated by gender salience and by mixed-gender friendships. Acknowledging the limitations of cross-sectional meditational models to causal inferences [50], the mediation analyses should be regarded as descriptive and exploratory, and were supplemented with additional analysis testing alternative mediation models.

### Hypotheses

We predicted that, compared to coeducational school students, single-sex school students would have higher gender salience (H_1_), fewer other-gender friends (H_2_), and higher anxiety in mixed-gender situations (H_3_). Prior studies have not found any consistent moderating effect of student gender on the differences related to single-sex schooling, and we expected the school differences to be similar in boys and girls (H_4_). Nevertheless, we included student gender as a potential moderator as most prior studies did [4,13]. Across the two samples, we predicted that differences between single-sex and coeducational school students may be more pronounced in the high school sample as the school differences may attenuate when all students were exposed to similar mixed-gender environments after graduation (H_5_). Also, we hypothesized that the potential school difference in mixed-gender anxiety was mediated by gender salience (H_6_) and mixed-gender friendships (H_7_).

## Study 1: High school sample

### Method

#### Participants

Participants were recruited from four local high schools (one all-girls, one all-boys and two coeducational) located in demographically diverse districts in Hong Kong. Participants included Form 1 to 6 (7^th^ to 12^th^ grade) students except for the all-girls school, from which Form 6 students were excluded due to the school’s arrangement for public exam preparation. Questionnaires were administered by a class teacher in each class. Participants completed measures on the dependent variables and reported their monthly family income, parental education and age, numbers of sisters and brothers, and ethnicity. This study was carried out in accordance with the recommendations and approval of the University of Hong Kong Human Research Ethics Committee (HKU HREC). Students’ assent and passive consent from parents or guardians were obtained before data collection. All subjects gave written informed consent in accordance with the Declaration of Helsinki.

A total of 2083 students participated in the study (participation rate: 84%). Twenty-four subjects were excluded from analyses due to missing or extreme data (11 coeducational school students did not indicate their gender and 13 students reported extremely unlikely values on demographic variables). The final sample (*N* = 2059, *M*_*age*_ = 15.78 years, *SD* = 2.03) consisted of 589 boys and 376 girls from the two coeducational schools, 416 boys from the all-boys school, and 678 girls from the all-girls school. This sample size had over 99% a priori power to detect small differences (.20 < *d* < .30) at *α* = .05, two-tailed [51]. Participants were mainly Chinese (89%). Among the four participating schools, the all-boys school and one coeducational school were in Band 2, while the all-girls school and the other coeducational school were in Band 3. All participating schools used Chinese as the teaching language. Some schools used spoken Cantonese and some used spoken Mandarin in class, but they used the same written language in formal printed materials. Therefore, the questionnaires were printed in Chinese. Consistent with prior research [3,4,8], participants from single-sex schools had parents with higher education level, *t*(2042) = -7.157, *p* < .001, and attended more academically excellent schools, *t*(2023) = -10.855, *p* < .001, unequal variances, than did participants from coeducational schools. The average parental education level of the sample (i.e., between junior secondary to senior secondary) was similar to a representative sample of over 9000 students from local high schools, one from each of the 19 districts in Hong Kong [52]. Detailed participant characteristics are presented in Table 1.

**Table 1:** Participant characteristics by school type and student gender (Study 1: High school sample).

Demographic variables ^a^	Coeducational schools	Single-sex schools		Male	Female	
	*M (SD)*	*M (SD)*	*p*	*M (SD)*	*M (SD)*	*p*
Age (Range: 11–22)	16.58 (2.13)	15.07 (1.63)	< .001	15.83 (2.10)	15.73 (1.96)	.242
Monthly family income (HKD) ^b^ (Range: 0–1000000)	33292 (55082)	38594 (63227)	.044	44756 (77211)	27865 (33424)	< .001
Parental education attainment (Range: 1–6) ^c^	3.64 (.93)	3.94 (.97)	< .001	3.90 (.98)	3.70 (.93)	< .001
Parents' average age (Range: 30–67)	46.26 (5.56)	45.84 (5.70)	.095	46.25 (5.71)	45.84 (5.56)	.099
Number of brothers (Range: 0–5)	.61 (.77)	.55 (.70)	.058	.47 (.65)	.67 (.79)	< .001
Number of sisters (Range: 0–5)	.60 (.81)	.57 (.78)	.401	.52 (.72)	.63 (.86)	.002
School banding (Range: 2–3)	2.39 (.49)	2.62 (.49)	< .001	2.20 (.40)	2.80 (.40)	< .001

^a^ Variables in this table were included as covariates for the high school sample.

^b^ 1.00 HKD ≈ 0.13 USD.

^c^ 1 = no schooling/pre-primary, 2 = primary, 3 = junior secondary, 4 = senior secondary, 5 = post-secondary, 6 = postgraduate.

#### Gender salience measure

Gender salience was measured by McGuire et al.’s method [53]. This measure has been shown to be associated with or affected by gender composition [27,53]. Participants were asked to give three short responses to each of these two questions: “Tell me what you are” and “Tell me what you are not”. They were instructed to write down whatever they could think of immediately. The responses were then coded as “gender-related” or “non-gender-related”. Table 2 shows examples of this coding scheme. Answers were first coded separately by two coders. Inter-rater reliability was high for all answers (all *κ* > .93). Disagreements were then resolved by discussions between the two coders. The total number of gender-related answers across the two questions was summed to indicate gender salience.

**Table 2:** Coding scheme of the gender salience measure adapted from McGuire et al.’s method [53].

Categories of codes	Definition	Examples
0 = Non-gender-related answer	The answer is completely irrelevant to gender.	“I am a student.” “I am not a teacher.”
1 = Gender-related answer	Gender is directly mentioned in the answer, or the answer is implicitly related to gender.	"I am a girl." "I am a boy." “I am a man.” “I am a daughter.” “I am not a woman.” “I am not a father.”

#### Percentages of other-gender friends and close friends measure

To provide an indicator of mixed-gender friendships, participants reported the percentage of their same-gender friends and the percentage of their same-gender close friends. Clear definitions were given to the participants to differentiate “friend” and “close friend” (i.e., “friend” referred to “someone whom you know the name and go out on activities in group but not alone”; “close friend” referred to “someone whom you know the name, go out on activities in group or alone and share your emotional feeling with, and he/she also shares his/her emotional feelings with you, and provides honest feedback to you”). The responses were reverse-coded to indicate the percentages of other-gender friends and close friends.

#### Mixed-gender anxiety measure

The Dating Anxiety Scale for Adolescents (DAS-A) [31] was modified to measure mixed-gender anxiety. The original 21-item scale was designed to measure distress and concerns about negative evaluation in dating as well as non-dating mixed-gender situations. The first author reviewed the items with a group of university students. No item was deemed culturally inappropriate. We were interested in both dating anxiety and general mixed-gender anxiety, but because many high school participants were unlikely to have had dating experience, 10 items involving actual dating scenarios were excluded, resulting in a total of 11 test items, of which 3 items measured Fear of Negative Evaluation (e.g., “I worry that I may not be attractive to people of the opposite sex”), 4 items measured Social Distress in Potential Romantic Relationship (e.g., “I become tense and jittery when I feel that someone of the opposite-sex is checking me out”), and 4 items measured Social Distress in Mixed-gender Groups (e.g., “It takes me a long time to feel comfortable when I am in a group of both males and females.”). Three filler items (e.g., “I love to go to parties”) were included to provide a break from rating anxiety-related items. Responses were made on a five-point scale (ranging from 1 “completely disagree” to 5 “strongly agree”). Following Glickman and La Greca [31], scores were summed for each sub-scale. The internal reliability was good for total DAS-A (*α* = .913), Fear of Negative Evaluation (*α =* .797), Social Distress in Potential Romantic Relationship (*α* = .853), and Social Distress in Mixed-gender Groups (*α* = .824).

### Results

A series of 2 (school type: single-sex vs. coeducational) × 2 (student gender: male vs. female) ANCOVAs were conducted on gender salience, percentage of other-gender friends, percentage of other-gender close friends, total mixed-gender anxiety, and the three anxiety subscales (see Table 3). All the outcome variables in this study had skewness (ranging from .294 to 1.051) and kurtosis (ranging from .004 to .864) that were within acceptable ranges [54]. The estimated marginal means and standard errors of the outcome variables are shown in Table 4 (correlations among the study variables are presented in Table A in S1 File). The ANOVA results without covariates can be found in Table B in S1 File. Since results without control variables are considered less reliable, we will focus on the ANCOVA results. Mediation analyses were then conducted to explore whether school differences in mixed-gender anxiety were mediated by mixed-gender friendships and/or gender salience. All analyses controlled for family income, parental age, parental education, student age, number of brothers, number of sisters, and school banding.

**Table 3:** 2 × 2 (School type × Student gender) ANCOVA results (Study 1: High school sample).

Dependent variables	Main effects		Interactions
	School type ^a^	Student gender	School type × Student gender
**Mixed-gender anxiety**			
Total	SS > CE *F*(1, 2048) = 11.64, *p =* .001, *d* = .15	M > F *F*(1, 2048) = 10.85, *p* = .001, *d* = .15	*F*(1, 2048) = .51, *p* = .474
Fear of Negative Evaluation	SS > CE *F*(1, 2048) = 8.30, *p* = .004, *d* = .13	M > F *F*(1, 2048) = 19.55, *p* < .001, *d* = .20	*F*(1, 2048) = .43, *p* = .512
Social Distress in Potential Romantic Relationship	SS > CE *F*(1, 2048) = 6.37, *p* = .012, *d* = .11	M > F *F*(1, 2048) = 8.05, *p* = .005, *d* = .13	*F*(1, 2048) = 2.40, *p* = .122
Social Distress in Mixed-gender Groups	SS > CE *F*(1, 2048) = 13.41, *p* < .001, *d* = .16	*F*(1, 2048) = 3.41, *p* = .065	*F*(1, 2048) = .13, *p* = .715
**Gender salience**	SS > CE *F* (1, 2048) = 29.36, *p* < .001, *d* = .24	F > M *F*(1, 2048) = 13.79, *p <* .001, *d* = .16	*F*(1, 2048) = .16, *p* = .694
**Percentage of other-gender close friends**	CE > SS *F*(1, 2048) = 33.98, *p* < .001, *d* = .26	*F*(1, 2048) = .52, *p* = .470	*F*(1, 2048) = .70, *p* = .402
**Percentage of other-gender friends**	CE > SS *F*(1, 2048) = 143.78, *p* < .001, *d* = .53	F > M *F*(1, 2048) = 6.29, *p* = .012, *d* = .11	*F*(1, 2048) = 2.06, *p* = .151

^a^ SS denotes single-sex school; CE denotes coeducational school.

**Table 4:** Estimated marginal means and standard errors of the outcome variables (Study 1: High school sample).

Outcome variables	Coeducational schools						Single-sex schools					
	Male (*n* = 589)		Female (*n* = 376)		Total (*n* = 965)		Male (*n* = 416)		Female (*n* = 678)		Total (*n* = 1094)	
	*M*	*SE*	*M*	*SE*	*M*	*SE*	*M*	*SE*	*M*	*SE*	*M*	*SE*
Gender salience	.92	.05	1.15	.06	1.03	.04	1.21	.07	1.49	.06	1.35	.04
Percentage of other-gender friends	31.29	.90	35.96	1.10	33.63	.73	20.50	1.27	21.93	1.05	21.22	.67
Percentage of other-gender close friends	31.61	1.26	29.05	1.54	30.33	1.02	21.84	1.77	21.94	1.47	21.89	.94
Mixed-gender anxiety—Total	29.13	.43	27.58	.53	28.35	.35	31.23	.61	28.90	.51	30.06	.33
Mixed-gender anxiety—Fear of Negative Evaluation	8.08	.13	7.39	.16	7.73	.11	8.63	.19	7.72	.16	8.17	.10
Mixed-gender anxiety—Social Distress in Potential Romantic Relationship	11.18	.19	10.83	.23	11.00	.15	12.08	.26	11.00	.22	11.54	.14
Mixed-gender anxiety—Social Distress in Mixed-gender Groups	9.88	.17	9.37	.21	9.62	.14	10.52	.24	10.17	.20	10.35	.13

#### Gender salience

Single-sex school students gave more gender-related answers to the questions “tell me what you are” and “tell me what you are not” than coeducational school students, *p* < .001, *d* = .24, meaning that single-sex school students were more gender-salient than coeducational school students, supporting H_1_. There was also a main effect of student gender, with female students being more gender-salient than male students, *p* < .001, *d* = .16. Supporting H_4,_ no interaction effect with student gender was found.

#### Percentages of other-gender friends and close friends

Consistent with H_2_, coeducational school students reported having higher percentages of other-gender friends (*p* < .001, *d* = .53) and other-gender close friends (*p* < .001, *d* = .26) than single-sex school students. Also, female students reported a higher percentage of other-gender friends than male students (*p* = .012, *d* = .11). There was no main effect of student gender in the percentage of other-gender close friends. Supporting H_4,_ there was no interaction effect with student gender.

#### Mixed-gender anxiety

Consistent with H_3_, compared to coeducational school students, single-sex school students reported higher levels of total mixed-gender anxiety, *p* = .001, *d* = .15, Fear of Negative Evaluation, *p* = .004, *d* = .13, Social Distress in Potential Romantic Relationship, *p* = .012, *d* = .11, and Social Distress in Mixed-gender Groups, *p* < .001, *d* = .16, even when students’ demographic characteristics were controlled for. There were also main effects of student gender, with male students reporting more total mixed-gender anxiety, *p* = .001, *d* = .15, more Fear of Negative Evaluation, *p* < .001, *d* = .20, and more Social Distress in Potential Romantic Relationship, *p* = .005, *d* = .13, than female students. Supporting H_4,_ there was no interaction effect with student gender.

#### Mediations

**Fig 1:**
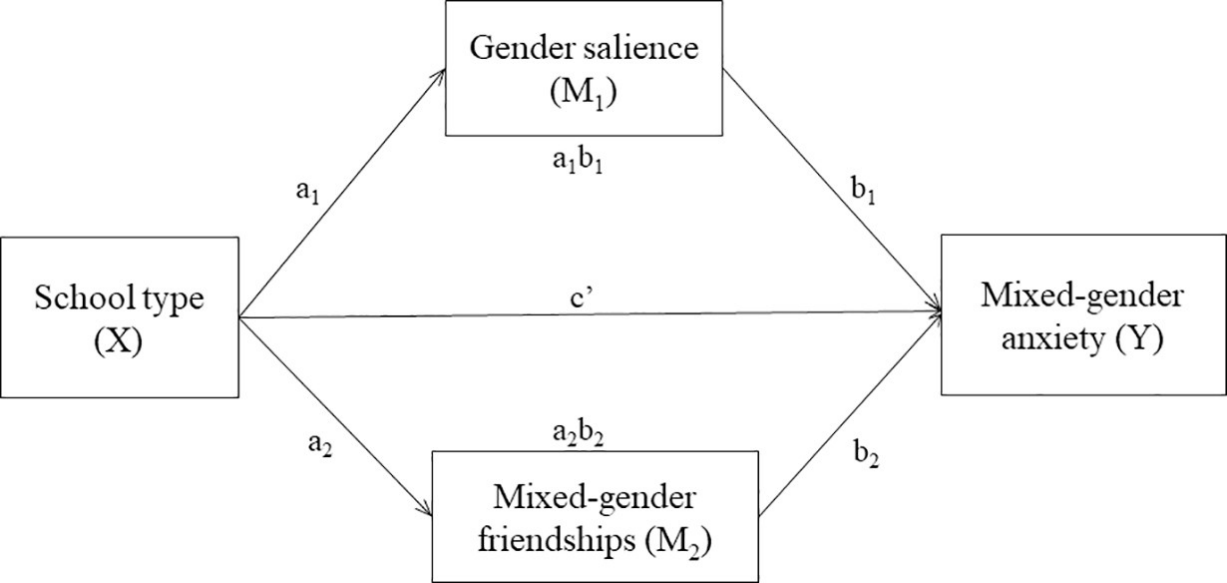
Generic mediation model. In the mediation model, a_1_b_1_ denotes the indirect effect (i.e., mediation) of gender salience, a_2_b_2_ denotes the indirect effect of mixed-gender friendships, and c’ denotes the direct effect of school type on mixed-gender anxiety.

Mediation analyses using 10,000 bootstrap samples were conducted by the SPSS macro PROCESS [55] to test whether the association between single-gender schooling (X) and students’ mixed-gender anxiety took place through the mediators (M), gender salience and/or mixed-gender friendships. Separate analyses were conducted for the different forms of mixed-gender anxiety as the dependent variable (Y). Monthly family income, parental education, parental age, student age, school banding, number of brothers and number of sisters were entered as covariates. Gender salience and mixed-gender friendships entered the mediation model as two simultaneous mediators (see Fig 1 for the generic mediation model and Table 5 for the results). There were significant indirect effects (i.e., mediation) of percentage of other-gender friends on all forms of mixed-gender anxiety, supporting H_7_. There were also significant indirect effects of percentage of other-gender close friends on all forms of mixed-gender anxiety except for Fear of Negative Evaluation. However, contrary to H_6,_ gender salience had no significant indirect effects on mixed-gender anxiety.

**Table 5:** Mediation results (Study 1: High school sample).

	Total effect (*p*)^c^	Model 1 ^a^			Model 2 ^b^		
		Direct effect (c’) (*p*) ^c^	Indirect effect ^c^		Direct effect (c’) (*p*) ^c^	Indirect effect^c^	
			Gender Salience (a_1_b_1_) [LLCI, ULCI]	Percentage of other-gender friends (a_2_b_2_) [LLCI, ULCI]		Gender Salience (a_1_b_1_) [LLCI, ULCI]	Percentage of other-gender close friends (a_2_b_2_) [LLCI, ULCI]
**Mixed-gender anxiety**							
Total	.1506 (.002)*	.0409 (.420)	.0057 [-.0067, .0198]	.1040 [.0755, .1376]*	.1131 (.024)*	.0058 [-.0067, .0200]	.0317 [.0178, .0508]*
Fear of Negative Evaluation	.1149 (.020)*	.0463 (.365)	.0110 [-.0018, .0263]	.0576 [.0318, .0878]*	.0914 (.069)	.0110 [-.0022, .0265]	.0125 [-.0004, .0285]
Social Distress in Potential Romantic Relationship	.1148 (.021)*	.0002 (.997)	.0106 [-.0016, .0249]	.1039 [.0750, .1376]*	.0683 (.173)	.0107 [-.0017, .0255]	.0358 [.0210, .0564]*
Social Distress in Mixed-gender Groups	.1677 (.001)*	.0669 (.186)	-.0054 [-.0192, .0067]	.1062 [.0769, .1409]*	.1412 (.005)*	-.0053 [-.0190, .0073]	.0318 [.0175, .0505]*

^a^ Model 1: X = School type (CE = 0, SS = 1); M_1_ = Gender salience, M_2_ = Percentage of other-gender friends; Y = Mixed-gender anxiety.

^b^ Model 2: X = School type (CE = 0, SS = 1); M_1_ = Gender salience, M_2_ = Percentage of other-gender close friends; Y = Mixed-gender anxiety.

^c^ In mediation models, the total effect refers to the association between the dependent variable (Y) and the independent variable (X), in which the indirect effect indicates the association mediated by the mediator (M) and the direct effect indicates the remaining association when the mediator is taken away. Coefficients were calculated with standardized values of gender salience, mixed-gender anxiety and percentage of other-gender friends and close friends. School type was not standardized because it is a dichotomous variable.

* denotes statistical significance.

To examine whether the direction of mediation effects was reversible, we also tested alternative mediation models (see Figure A in S1 File), with mixed-gender anxiety as the mediator and mixed-gender friendships as the dependent variable. School type was always set to be the independent variable because it was already fixed before the measurements of mixed-gender anxiety and mixed-gender friendships. Results of the alternative mediation analyses (see Table C in S1 File) showed that there were significant indirect effects of all forms of mixed-gender anxiety on percentages of other-gender friends and other-gender close friends.

Study 1 showed that adolescent students currently gender-segregated at school scored higher on gender salience, had fewer mixed-gender friendships, and reported higher anxiety in mixed-gender interactions. The higher mixed-gender anxiety related to reduced mixed-gender friendships but not increased gender salience. What remains unknown is whether these findings would also be found in students who have left the gender-segregated school environment. It may be that differences between single-sex and coeducational school students remain but attenuate when all students are exposed to similar mixed-gender environments after graduation. To investigate how single-sex and coeducational school students differ at different stages in life, Study 2 tested gender salience, mixed-gender friendship and mixed-gender anxiety in a college sample.

## Study 2: College sample

### Method

#### Participants

Four hundred and eighty-three participants were recruited from a large university in Hong Kong through advertisements posted in campus and halls, mass emails through departments and faculties, and snowballing. Testing took place in a laboratory. Participants completed the gender salience measure, the mixed-gender anxiety scale and reported their demographic background. All measures were presented in their original English language as English is the medium of instruction for tertiary education in Hong Kong. This study was carried out in accordance with the recommendations and approval of the University of Hong Kong Human Research Ethics Committee (HKU HREC). All subjects gave written informed consent in accordance with the Declaration of Helsinki. Parental consent was not required by the HKU HREC for this sample.

Participants reported the name, type (single-sex or coeducational) and banding of the high schools they had attended. School banding was averaged if participants had attended more than one high school (only 4.1% of the total sample). Participants also reported their college year, total parental income and parental education, numbers of sisters and brothers, faculty, and sexual orientation. Students’ faculty was coded as male-dominated, female-dominated, or gender-balanced based on enrolment statistics [56]. Sexual orientation was assessed using the adapted Klein Sexual Orientation Grid (KSOG) [57]. Participants rated their sexual orientation in the past 12 months on four aspects, namely attraction, behavior, fantasy, and identity, from 0 (other-sex only) to 6 (same-sex only). The number of participants was roughly balanced by school type and student gender. Participants who had switched between single-sex and coeducational schools were excluded from analysis (*n* = 27). The final sample consisted of 456 graduates (239 females; 52.4%) who had attended 182 different high schools. These graduates either attended single-sex schools (*n* = 207) or coeducational schools (*n* = 249) for high school education. This sample size had over 80% a priori power to detect small differences (.26 < *d* < .30) at *α* = .05, two-tailed [51]. The sample was almost exclusively Chinese (97.6%) and aged 19.53 years on average. The mean parental income of the sample (HKD42632) suggested that the sample was demographically similar to undergraduate students from several local universities (e.g., [58–60]).

Table 6 presents detailed participant characteristics by school type and student gender. Consistent with prior research [3,4,8], participants from single-sex schools had parents with higher education level, *t*(454) = 3.48, *p* = .001, and attended more academically excellent schools, *t*(387) = 4.26, *p* < .001, unequal variances, than did participants from coeducational schools.

**Table 6:** Participant characteristics by school type and student gender (Study 2: College sample).

Demographic variables	Coeducational schools	Single-sex schools		Male	Female	
	*M (SD)*	*M (SD)*	*p*	*M (SD)*	*M (SD)*	*p*
Age (Range: 17–25)	19.59 (1.51)	19.45 (1.43)	.311	19.61 (1.50)	19.45 (1.45)	.259
Monthly parental income (HKD)^a^ (Range: 0–1000000)	40409 (69136)	45307 (36051)	.358	39958 (31822)	45061 (72010)	.337
Parental education attainment ^b^ (Range: 1–6)	4.00 (1.13)	4.34 (.95)	.001	4.08 (1.01)	4.22 (1.11)	.150
Number of brothers (Range: 0–4)	.42 (.59)	.35 (.51)	.187	.37 (.57)	.41 (.54)	.428
Number of sisters (Range: 0–4)	.56 (.74)	.47 (.65)	.156	.49 (.63)	.54 (.76)	.436
School banding (Range: 1–3)	1.18 (.44)	1.05 (.23)	< .001	1.15 (.42)	1.09 (.31)	.085
Sexual attraction ^c^ (Range: 0–6)	1.26 (1.72)	1.34 (1.68)	.609	1.33 (1.74)	1.27 (1.67)	.688
Sexual behavior ^c^ (Range: 0–6)	.90 (1.71)	1.03 (1.78)	.400	.93 (1.71)	.99 (1.78)	.709
Sexual fantasy ^c^ (Range: 0–6)	.98 (1.66)	1.01 (1.66)	.829	.91 (1.69)	1.06 (1.62)	.334
Sexual identity ^c^ (Range: 0–6)	1.17 (1.87)	1.13 (1.58)	.794	1.12 (1.76)	1.18 (1.73)	.694
General social anxiety ^d^ (Range: 20–80)	50.16 (13.10)	49.70 (12.49)	.703	49.17 (13.15)	50.66 (12.49)	.215

^a^ 1.00 HKD ≈ 0.13 USD.

^b^ 1 = no schooling/pre-primary, 2 = primary, 3 = junior secondary, 4 = senior secondary, 5 = post-secondary, 6 = postgraduate.

^c^ 0 = other sex only, 1 = other sex mostly, 2 = other sex somewhat more, 3 = both sex equally, 4 = same sex somewhat more, 5 = same sex mostly, 6 = same sex only.

^d^ Variables in this table were included as covariates in all analyses, except for general social anxiety which was included as a covariate only in analyses involving mixed-gender anxiety for the college sample.

#### Gender salience measure

Gender salience was measured by the same method used in Study 1. The inter-rater reliability was good for all answers (*κ* ranging from .87 to .99).

#### Percentage of other-gender close friends measure

Percentage of other-gender close friends was measured by the same method used in Study 1. Percentage of other-gender friends was not assessed in the college sample.

#### Mixed-gender anxiety measure

As in Study 1, participants reported their mixed-gender anxiety on the modified dating anxiety scale (DAS-A) [31]. In contrast to Study 1, participants in Study 2 completed the full 21-item version of DAS-A because dating experience was common in the college sample (93.4% of the participants reported some sort of dating experience; see list of dating experience items from the Dating History Questionnaire [61] in Table D in S1 File). Those who had never dated before were asked to imagine how they would feel and behave in the described situations. For this full scale, ten items measured Fear of Negative Evaluation, seven items measured Social Distress in Dating Situations, four items measured Social Distress in Mixed-gender Groups, and five filler items provided a break from rating anxiety-related items. The internal consistency was good for total DAS-A (*α* = .95), Fear of Negative Evaluation (*α* = .92), Social Distress in Dating (*α* = .89), and Social Distress in Mixed-gender Groups (*α* = .80). Since our focus was not only on dating anxiety and only 2.6% of the college participants reported having no heterosexual attraction at all, we included all participants in the analysis of mixed-gender anxiety and controlled for sexual orientation.

To rule out the effect of anxiety not specific to mixed-gender situations, we additionally controlled for social anxiety in analyses involving mixed-gender anxiety, as did Glickman and La Greca [31]. Social anxiety was measured by the Social Anxiety Scale for adolescents (SAS-A) [62] which contained 18 items that reflected general anxiety felt in social situations (e.g., “I feel shy around people I don’t know”). Each item was rated on a 5-point scale ranging from 1 (not at all) to 5 (all the time). The total social anxiety score was the sum of all items. Reliability of the SAS-A was high (*α* = .93).

### Results

A series of 2 (school: single-sex vs. coeducational) × 2 (student gender: male vs. female) ANCOVAs were conducted on gender salience, percentage of other-gender close friends, total mixed-gender anxiety and the three anxiety subscales (see Table 7). All the outcome variables had skewness (ranging from .040 to 1.235) and kurtosis (ranging from .488 to .670) that were within acceptable ranges [54]. The estimated marginal means and standard errors of the outcome variables are shown in Table 8 (correlations among the study variables are presented in Table E in S1 File). The ANOVA results without covariates can be found in Table F in S1 File. Mediation analyses were conducted to explore whether school differences in mixed-gender anxiety were mediated by mixed-gender friendships and/or gender salience. All analyses controlled for parental income, parental education, number of brothers, number of sisters, school banding, the four dimensions of sexual orientation, faculty, and student age; the analyses on mixed-gender anxiety also controlled for social anxiety.

**Table 7:** 2 × 2 (School type × Student gender) ANCOVA results (Study 2: College sample).

Dependent variables	Main effects		Interactions
	School type^a^	Student gender	School type × Student gender
**Mixed-sex anxiety**			
Total	SS > CE *F*(1, 440) = 6.92, *p* = .009, *d* = .25	M > F *F*(1, 440) = 5.43, *p* = .020, *d* = .22	*F*(1, 440) = .07, *p* = .798
Fear of Negative Evaluation	*F*(1, 440) = 2.17, *p* = .141	M > F *F*(1, 440) = 7.16, *p* = .008, *d* = .25	*F*(1, 440) = .79, *p* = .375
Social Distress in Dating	SS > CE *F*(1, 440) = 7.36, *p* = .007, *d* = .26	*F*(1, 440) = 2.31, *p* = .129	*F*(1, 440) = .63, *p* = .427
Social Distress in Mixed-gender Groups	SS > CE *F*(1, 440) = 7.37, *p* = .007, *d* = .26	*F*(1, 440) = .48, *p* = .489	*F*(1, 440) = 3.20, *p* = .074
**Gender salience**	*F*(1, 441) = .25, *p* = .616	*F*(1, 441) = .04, *p* = .840	*F*(1, 441) = .37, *p* = .545
**Percentage of other-gender close friends**	CE > SS *F*(1, 441) = 24.80, *p* < .001, *d* = .47	M > F *F*(1, 441) = 7.96, *p* = .005, *d* = .27	*F*(1, 441) = .05, *p* = .829

^a^ SS denotes Single-sex school; CE denotes Coeducational school.

**Table 8:** Estimated marginal means and standard errors of the outcome variables (Study 2: College sample).

Outcome variables	Coeducational schools						Single-sex schools					
	Male (*n* = 120)		Female (*n* = 129)		Total (*n* = 249)		Male (*n* = 97)		Female (*n* = 110)		Total (*n* = 207)	
	*M*	*SE*	*M*	*SE*	*M*	*SE*	*M*	*SE*	*M*	*SE*	*M*	*SE*
Gender salience	.64	.08	.61	.08	.62	.06	.55	.09	.61	.08	.58	.06
Percentage of other-gender close friends	35.88	2.25	29.74	2.15	32.81	1.55	24.69	2.50	17.57	2.34	21.13	1.71
Mixed-gender anxiety—Total	61.46	1.03	58.68	.98	60.07	.71	64.00	1.14	61.77	1.07	62.88	.78
Mixed-gender anxiety—Fear of Negative Evaluation	30.45	.59	29.33	.56	29.89	.41	31.88	.65	29.70	.61	30.79	.45
Mixed-gender anxiety—Social Distress in Dating	20.83	.43	19.81	.41	20.32	.30	21.70	.48	21.36	.44	21.53	.32
Mixed-gender anxiety—Social Distress in Mixed-gender Groups	10.18	.25	9.54	.24	9.86	.17	10.43	.28	10.70	.26	10.56	.19

#### Gender salience

In contrast to Study 1, there were no main effects of school type or student gender and no interaction effects on gender salience. Therefore, H_1_ was not supported.

#### Percentage of other-gender close friends

There was a main effect of school type, with coeducational school students reporting a larger percentage of other-gender close friends than single-sex school students, *p* < .001, *d* = .47, supporting H_2_. There was also a main effect of student gender, with male students reporting a larger percentage of other-gender close friends than female students (*p* = .005, *d* = .27). Consistent with H_4_, there was no interaction effect with student gender.

#### Mixed-gender anxiety

Single-sex school students reported higher levels of total mixed-gender anxiety (*p* = .009, *d* = .25), Social Distress in Dating (*p* = .007, *d* = .26), and Social Distress in Mixed-gender Groups (*p* = .007, *d* = .26) than coeducational school students. There was no main effect of school in Fear of Negative Evaluation. Therefore, H_3_ was largely supported. Male students reported higher levels of total mixed-gender anxiety (*p* = .020, *d* = .22) and Fear of Negative Evaluation (*p* = .008, *d* = .25) than female students. There were no main effects of student gender in Social Distress in Dating and Social Distress in Mixed-gender Groups. Consistent with H_4_, there were no interaction effects with student gender in all forms of mixed-gender anxiety.

#### Supplementary analysis: Did school differences depend on college year?

Comparing across the two samples, the differences between single-sex school students and coeducational school students were more pronounced in the high school sample, supporting H_5_. For example, gender salience and fear of negative evaluation differed between single-sex and coeducational school students only in the high school sample.

We further conducted a series of “School type (single-sex vs. coeducational) × Student gender (male vs. female) × College year (first year vs. non-first year)” ANCOVAs on the college sample (see Table G in supplementary materials) to test for potential college year effects. Results showed no main effect of college year or any interaction involving college year.

#### Mediations

As in Study 1, mediation analyses were conducted using PROCESS with 10,000 bootstrap samples and the same mediation model, except that for Study 2, the covariates were parental income, parental education, number of brothers, number of sisters, school banding, the four dimensions of sexual orientation, faculty, student age, and social anxiety. Each form of mixed-gender anxiety was analyzed separately (see Table 9). Percentage of other-gender close friends mediated the school differences in total mixed-gender anxiety, Social Distress in Dating, and Social Distress in Mixed-gender Groups, but not Fear of Negative Evaluation. Thus, H_7_ was partially supported. As in Study 1, there were no significant indirect effects of gender salience on either total or any particular form of mixed-gender anxiety. Alternative mediation models were also conducted (see Figure A in S1 File for the generic alternative mediation model and Table H for the results). Results showed significant indirect effects of total mixed-gender anxiety, Social Distress in Dating and Social Distress in Mixed-gender Groups on the percentage of other-gender close friends.

**Table 9:** Mediation results (Study 2: College sample).

Dependent variables ^a^	Total effect (*p*) ^b^	Direct effect (c’) (*p*) ^b^	Indirect effect ^b^	
			Gender Salience (a_1_b_1_) [LLCI, ULCI]	Percentage of other-gender close friends (a_2_b_2_) [LLCI, ULCI]
**Mixed-gender anxiety**				
Total	.1723 (.009)*	.1173 (.081)	.0009 [-.0035, .0160]	.0541 [.0224, .0995]*
Fear of Negative Evaluation	.1001 (.168)	.0715 (.337)	.0013 [-.0038, .0195]	.0273 [-.0072, .0720]
Social Distress in Dating	.2027 (.006)*	.1220 (.101)	.0010 [-.0040, .0179]	.0797 [.0383, .1357]*
Social Distress in Mixed-gender Groups	.2028 (.005)*	.1570 (.034)*	-.0009 [-.0150, .0035]	.0467 [.0149, .0924]*

^a^ Mediation model: X = School type (CE = 0 SS = 1); M_1_ = Gender salience, M_2_ = Percentage of other-gender close friends; Y = Mixed-gender anxiety.

^b^ In mediation models, the total effect refers to the association between the dependent variable (Y) and the independent variable (X), in which the indirect effect indicates the association mediated by the mediator (M) and the direct effect indicates the remaining association when the mediator is taken away. Coefficients were calculated with standardized values of gender salience, mixed-gender anxiety and percentage of other-gender friends. School type was not standardized because it is a dichotomous variable.

* denotes statistical significance.

## Discussion

Schooling experience represents an important developmental influence. Apart from fostering academic skills, public education should also prepare students for mixed-gender workplaces, families, and citizenry [2]. Stakeholders of single-sex schooling have therefore been concerned about the impact of gender-segregated schooling on social development, especially the extent to which students can handle mixed-gender situations with ease. However, research on single-sex schooling has focused on academic outcomes and provides little knowledge on its social outcomes. We provide the first systematic comparison of students from single-sex and coeducational schools on gender salience and mixed-gender anxiety in a high school sample and a college sample. Even when demographic characteristics were controlled, our results supported the hypotheses that single-sex school students had higher gender salience (H_1_) in the high school sample, and that single-sex school students had fewer other-gender friends (H_2_) and higher mixed-gender anxiety (H_3_) in both high school and college samples. The hypothesis that such school differences were similar between boys and girls (H_4_) was also supported. More outcomes were found to differ by school type in the high school sample than in the college sample, providing support for H_5_. Moreover, the association between school type and mixed-gender anxiety was mediated by mixed-gender friendships in both samples (H_7_ supported), but not by gender salience (H_6_ not supported). These results illuminate the gender cognition and social development of students and have implications for school policies.

### Mixed-gender anxiety

Mixed-gender anxiety may affect people’s adjustments in both romantic situations and non-romantic situations. Dating anxiety is more relevant to romantic situations and mainly affects heterosexuals whereas general mixed-gender anxiety is more relevant to non-romantic situations and affects individuals of any sexual orientation. The scale we used to measure mixed-gender anxiety was analyzed in different subscales given specific names to indicate whether the items concerned romantic (i.e., the Social Distress in Dating subscale in the college sample and the Social Distress in Potential Romantic Relationship in the high school sample) or general non-romantic situations (i.e., the Fear of Negative Evaluation and the Social Distress in Mixed-gender Groups subscales). Participants reported the level of anxiety in various mixed-gender situations such as actual dating (in the college sample), meeting a potential dating partner and casual get-togethers. All groups of participants reported moderate levels of anxiety, reflecting the centrality of mixed-gender relationship that adolescents and young adults attach to themselves [1,29,32,33]. However, male students reported greater mixed-gender anxiety in the form of Fear of Negative Evaluation (in both samples) and Social Distress in Potential Romantic Relationship (in the high school sample). Prior studies have also found that males reported greater anxiety towards mixed-gender romance and friendships [31,36,63]. The direction and size of the gender differences (*d* ranging from .13 to .25) were similar to the gender difference in anxiety in mixed-gender groups found in a slightly younger, adolescent sample [31], suggesting good validity of this scale in the current sample. This gender difference may be related to the greater social expectation for men than women to take initiative and make an impression in mixed-gender interactions [36,63].

More importantly, compared to coeducational school students, current single-sex school students scored higher on all forms of mixed-gender anxiety, and graduates from single-sex schools scored higher on anxiety in dating situations and casual mixed-gender groups, even after controlling for general social anxiety. Results were in line with the evidence of more negative marriage outcomes in middle-aged graduates of single-sex schools [11,42] and one small uncontrolled study that found lower mixed-gender friendship satisfaction in 10^th^ grade students of single-sex schools [43]. We further showed that the difference in mixed-gender anxiety was significant even after controlling for demographic characteristics that often cloud comparisons of single-sex and coeducational school students, and that this difference existed above and beyond general social anxiety.

The differences in mixed-gender anxiety between single-sex and coeducational schools (*d*s ranging from .11 to .26) were small by statistical convention [51], but were at least as large as half of the difference found between older and younger students differing by one grade in high school [31]. Moreover, it is intriguing that the effect of school did not interact with student gender, suggesting that both male and female students from single-sex schools experienced more mixed-gender anxiety.

Because the social outcomes of single-sex school graduates may change after they immerse themselves into a coeducational environment (e.g., [64]), we conducted supplementary analyses comparing first-year students and senior students to test for potential college year effects. However, it appeared that the differences between single-sex and coeducational school graduates in mixed-gender anxiety and friendships did not change throughout the college years, implying that the school type effects were long lasting.

### Potential mechanisms associated with differences between single-sex and coeducational school students

Besides mixed-gender anxiety, mixed-gender friendships and gender salience were compared between school types. They were also tested as potential mediators of the school differences in mixed-gender anxiety. As expected based on the finding that same-gender peer preference remains strong throughout the lifespan [1], both coeducational and single-sex school students reported that only a minority of their friends, regardless of close friends or not, were of a different gender. In particular, single-sex school students reported having a smaller percentage of other-gender friends than coeducational school students and this tendency appeared to be remarkably stable across the high school and college samples, with coeducational school students and single-sex school students reporting roughly 30% and 20% of their friendships being mixed-gender, respectively.

Consistent with the negative correlation between mixed-gender friendships and mixed-gender anxiety [34,35], mixed-gender friendships mediated the school differences in mixed-gender anxiety in both high school and college samples, suggesting that having fewer other-gender friends may be a possible reason why single-sex school students felt more mixed-gender anxiety. There were significant indirect effects in the alternative mediation models for both samples, meaning that single-sex schooling may also lead to reduced mixed-gender friendships by heightening mixed-gender anxiety. These bi-directional mediations were consistent with the view that mixed-gender anxiety and poor mixed-gender social skills or relationships may escalate in a vicious cycle [29]. However, magnitudes of the indirect effects in the alternative mediation models (see the absolute values of *ab* in Tables C and H in S1 File) were consistently smaller than those in the original mediation models (see Tables 5 and 9), suggesting that the mediation effects were stronger in the path from mixed-gender friendships to mixed-gender anxiety than vice versa.

The finding that gender salience was higher in current single-sex school students supported the speculations against single-sex schooling [2,4]. To our knowledge, this is the first empirical evidence showing directly a difference in gender salience between students from single-sex and coeducational schools. This difference, however, was found only in the high school sample, suggesting that any effect of gender-segregated schooling on this variable disappears within a few years upon departure from the gender-segregated environment. This finding did not preclude the possibility that certain characteristics of single-sex schools reduce gender salience (e.g., absence of other-gender peers), as suggested by proponents (e.g., [24]), but suggested that the characteristics that increase gender salience (e.g., the use of gender as a basis for segregation) may be more powerful. We hypothesized that gender salience would also mediate the school differences in mixed-gender anxiety because the awareness of gender appears to trigger an individual’s concerns about gender-related issues during interactions [16,46]. However, although gender salience showed the expected difference between school types, it was not a mediator of the school differences in mixed-gender anxiety.

### Policy implications

Mixed-gender interactions serve key functions in adolescents’ psychosocial development that are unique from same-gender interactions [29]. Anxiety in mixed-gender situations has negative social [29,31,34], psychological and physical effects [38–40]. People high on mixed-gender anxiety tend to avoid mixed-gender situations and be less happy about them, which may result in a disruption of the learning process of establishing functional mixed-gender relationships [29,31,32,34,37] and pose challenges for transition into adulthood. Although students in single-sex schools may not need to face interpersonal problems in mixed-gender situations at school, interactions in mixed-gender groups are inevitable at many points in life. For example, classes are rarely gender-segregated in college and many courses require students to form study groups randomly, where students will have to cooperate with both same- and other-gender peers in order to optimize their learning outcomes. In this case, mixed-gender anxiety may become an obstacle to getting better academic results. Moreover, mixed-gender anxiety may reduce students’ interest and motivation in pursuing their future study and/or career in the areas that are dominated by the other gender. When female students avoid science and engineering classes or when male students avoid nursing classes, the number of women in science and engineering jobs and the number of men in nursing jobs may be diminished, in turn exacerbating the problems when one gender is underrepresented in fields, such as inequity in earnings and stifled talent. Besides, as students are likely to meet their future partner during adolescence and early adulthood, mixed-gender anxiety may diminish the chance of building successful romantic relationships for heterosexual individuals.

Reduced exposure to mixed-gender interactions has been suggested to predispose adolescents to experience mixed-gender anxiety [34,35]. Consistent with this notion, we found students of single-sex schools reported having fewer other-gender friends in both high school and college samples. Also, single-sex school students reported higher levels of three different types of mixed-gender anxiety, two of which remained higher than coeducational school students even after leaving the gender-segregated environment. The effects were small. However, they were found in both male and female students, and remained in the college years. Moreover, the school type differences were mediated by mixed-gender friendships. These findings substantiated the concerns that the reduced opportunities for single-sex school students to engage in mixed-gender interactions may negatively affect their ability to deal with the other gender [2,8,10] and to adapt to society [9]. They suggested potential benefits for single-sex schools to increase mixed-gender activities early on in order to compensate for the inherently limited opportunities for mixed-gender interactions. Besides, for both male and female students, teaching more androgynous gender roles may be beneficial because higher masculinity has been found to correlate with higher discomfort with mixed-gender situations whereas androgynous men were more comfortable and confident than either masculine or undifferentiated men in these situations [63,65].

We did not find gender salience to be related to mixed-gender anxiety. This may imply that interventions for reducing mixed-gender anxiety should focus more on mixed-gender friendships instead of gender salience. However, it may be that gender salience mediates mixed-gender anxiety under specific circumstances, such as when coupled with low perceived competence in mixed-gender interactions. This finding also did not preclude the possibility for higher gender salience in single-sex school students to translate into greater gender-stereotyping, as predicted by the developmental intergroup theory [16]. However, currently it is difficult to make this conclusion because findings on how single-sex or coeducational school students differ on various aspects of gender-stereotyping and gender cognitions are mixed (cf. [25,26]; see meta-analysis by Pahlke et al. [4]). Nevertheless, the finding of higher gender salience in current students from single-sex schools itself may call for attention, as people with higher gender salience develop more rigid gender stereotypes and negativity towards the other gender [16,22,23].

### Limitations

As in many studies of single-sex schooling, random assignment was not possible, therefore there was no certainty that the observed differences between students from single-sex schools and coeducational schools were caused by gender segregation. Also, it would be impossible to control for all potentially confounding variables. However, by controlling for a multitude of key demographic variables, this study would be classified as one of the highly controlled studies on single-sex schooling [4]. Moreover, parents’ choice of schools is affected by a host of factors that are rarely gender-related [66], and the eventual school allocation involves a complex interplay between personal preference, academic ability, and procedural and logistical systems. It is thus questionable that students were self-selected into single-sex or coeducational schools based on pre-existing levels of the current outcomes (gender salience, mixed-gender friendships, and mixed-gender anxiety). Studies of parents’ and children’s reasons for choosing certain schools and a longitudinal study measuring the outcome variables prior to streaming into single-sex or coeducational schools will be helpful.

Mixed-gender anxiety and mixed-gender friendships were not measured at different time points, so we cannot rule out the possibility that the differences in mixed-gender friendship were mediated by mixed-gender anxiety. MacKinnon et al. [50] suggested regarding cross-sectional mediation as descriptive information because alternative causal explanations are possible. In consideration of this possibility, we also tested the alternative mediation models and the results suggested reciprocal influences between mixed-gender anxiety and mixed-gender friendships. While policy and ethical restrictions may make it difficult to use experimental paradigms to validate mediation relations such as those we hypothesized, qualitative studies may help to achieve this goal [50]. Regardless of causality, the descriptive differences in gender salience, mixed-gender anxiety, and mixed-gender friendships found between single-sex and coeducational school students and between high school and college students provide valuable data that inform an intense debate and intervention strategies.

The two samples each had limitations and strengths. Like many prior studies, the high school sample was limited by the inclusion of a few schools [67], but its sample size was large. The college sample was limited in the sense that it included high school graduates currently studying at a large university, and so may be more academically competent and of higher socioeconomic status than the general body of high school graduates. However, the generalizability of the college sample was increased by its inclusion of graduates from many different high schools. There was some evidence that the two samples were at least demographically similar to students from other high schools and universities in the same region (e.g., [52,58–60,68]). Besides, homogeneity of the sample may have the benefit of increasing control over sample characteristics [67,69]. Both samples were also relatively large compared to those in other single-sex schooling research on social outcomes, for which data cannot be provided by existing large-scale data sets (see studies included in [4]). Most importantly, the convergent evidence from the two samples, each with its own strengths and limitations, added to the reliability of the findings.

Results showed differences between students from single-sex and coeducational schools at both high school and college, suggesting that prior gender segregation had lasting effects on social outcomes. However, we were not able to show within-person changes. Longitudinal studies are needed to investigate these changes. Moreover, part of the study was based on self-reported mixed-gender anxiety. Well-controlled experimental or observational paradigms may be needed to better capture participants' experienced anxiety.

Transgender individuals may not fit into the current research on mixed-gender interaction and our study did not cover the dating anxiety of nonheterosexual individuals. Future studies could extend the scope of research to include more genders and sexual orientations and explore the type of anxiety which may be more relevant to transgender and nonheterosexual individuals.

### Conclusions

In response to stakeholders’ concerns about gender salience, mixed-gender relationships and mixed-gender anxiety of students deprived of mixed-gender experience at school [2,8–12], we found differences in gender salience, mixed-gender friendships and mixed-gender anxiety during high school and into the college years that favored coeducational schooling. High school students of single-sex schools were more gender salient, more anxious about mixed-gender situations and had fewer other-gender friends, and graduates of single-sex schools were still more anxious about mixed-gender interactions and had fewer other-gender friends. These early differences may have important implications for later marriage [11,42], academic, and career outcomes.

Results showed that single-sex schooling was associated with psychosocial outcomes both during and after the gender-segregated experience. Although stakeholders have focused on the short-term consequences of single-sex schooling [9], it will be important for policy makers to also consider its long-term consequences on students’ psychosocial development. The alleged academic benefits of single-sex schooling have recently been concluded as trivial or nonsignificant in several reviews and meta-analyses (e.g., [2–4]). It is timely for researchers to put more focus on evaluating the social consequences of single-sex schooling.

## Supporting information

S1 File Supplementary tables and figures. (DOC)
