# Supplementary material for: Students from single-sex schools are more gender-salient and more anxious in mixed-gender situations: Results from high school and college samples
Source: PLoS One. 2018 Dec 7;13(12):e0208707. doi: 10.1371/journal.pone.0208707 (PMC6286141; doi:10.1371/journal.pone.0208707)

**Table A.** Correlations for study variables (Study 1: High school sample).

|  | 1 | 2 | 3 | 4 | 5 | 6 | 7 | 8 | 9 | 10 | 11 | 12 | 13 | 14 | 15 | 16 |
| --- | --- | --- | --- | --- | --- | --- | --- | --- | --- | --- | --- | --- | --- | --- | --- | --- |
| 1. Student gender ^a^ |  | .230^**^ | .116^**^ | -.028 | -.081^**^ | -.057^**^ | -.125^**^ | -.039 | -.006 | -.142^**^ | -.108^**^ | -.036 | -.026 | .599^**^ | .139^**^ | .069^**^ |
| 1. School type ^b^ |  |  | .150^**^ | -.266^**^ | -.147^**^ | .032 | -.003 | .024 | .057^*^ | .044^*^ | .155^**^ | -.037 | -.372^**^ | .233^**^ | -.042 | -.019 |
| 1. Gender salience |  |  |  | -.046^*^ | -.029 | .025 | .034 | .041 | -.007 | -.056^*^ | -.024 | -.023 | -.088^**^ | .047^*^ | .028 | .024 |
| 1. Percentage of other-gender friends |  |  |  |  | .514^**^ | -.186^**^ | -.098^**^ | -.184^**^ | -.197^**^ | .083^**^ | .029 | -.046^*^ | .078^**^ | -.090^**^ | .073^**^ | .014 |
| 1. Percentage of other-gender close friends |  |  |  |  |  | -.109^**^ | -.038 | -.122^**^ | -.114^**^ | .095^**^ | .010 | -.020 | .039 | -.075^**^ | .023 | .019 |
| 1. Anxiety in mixed-gender situations - Total |  |  |  |  |  |  | .835^**^ | .935^**^ | .873^**^ | -.007 | -.056^*^ | .071^**^ | .051^*^ | -.019 | .012 | .054^*^ |
| 1. Anxiety in mixed-gender situations - Fear of Negative Evaluation |  |  |  |  |  |  |  | .727^**^ | .550^**^ | .013 | -.043^*^ | .078^**^ | .070^**^ | -.091^**^ | .001 | .020 |
| 1. Anxiety in mixed-gender situations - Potential Romantic Relationship |  |  |  |  |  |  |  |  | .722^**^ | -.023 | -.040 | .056^*^ | .041 | -.008 | .015 | .051^*^ |
| 1. Anxiety in mixed-gender situations - Social Distress in Mixed-gender Groups |  |  |  |  |  |  |  |  |  | -.004 | -.066^**^ | .058^**^ | .031 | .032 | .013 | .065^**^ |
| 1. Monthly family income |  |  |  |  |  |  |  |  |  |  | .122^**^ | -.002 | -.031 | -.122^**^ | -.003 | .018 |
| 1. Parents' education ^c^ |  |  |  |  |  |  |  |  |  |  |  | -.135^**^ | -.183^**^ | -.106^**^ | -.073^**^ | -.118^**^ |
| 1. Parents' age |  |  |  |  |  |  |  |  |  |  |  |  | .192^**^ | -.030 | .028 | .067^**^ |
| 1. Student age |  |  |  |  |  |  |  |  |  |  |  |  |  | -.012 | .022 | .014 |
| 1. School banding |  |  |  |  |  |  |  |  |  |  |  |  |  |  | .074^**^ | .076^**^ |
| 1. Number of brothers |  |  |  |  |  |  |  |  |  |  |  |  |  |  |  | .084^**^ |
| 1. Number of sisters |  |  |  |  |  |  |  |  |  |  |  |  |  |  |  |  |

*Note*: ^a^ 1 = male, 2 = female. ^b^ 0 = coeducational, 1 = single-sex. ^c^ 1 = no schooling/pre-primary, 2 = primary, 3 = junior secondary, 4 = senior secondary, 5 = post-secondary, 6 = postgraduate. * Correlation is significant at the .05 level (2-tailed). ** Correlation is significant at the .01 level (2-tailed).

**Table B.** 2 × 2 (School type × Student gender) ANOVA results (i.e., without covariates) of Study 1 (High school sample).

| Dependent variables | Main effects | | Interactions |
| --- | --- | --- | --- |
|  | School type | Student gender | School type × Student gender |
| **Mixed-gender anxiety** |  |  |  |
| Total | Single-sex > Coeducational  *F*(1, 2055) = 4.41, *p* = .036,  *d* = .09 | M > F  *F*(1, 2055) = 9.09, *p* = .003,  *d* = .13 | *F*(1, 2055) = .00, *p* = .953 |
| Fear of Negative Evaluation | *F*(1, 2055) = 1.42, *p* = .233 | M > F  *F*(1, 2055) = 33.42, *p* < .001,  *d* = .26 | *F*(1, 2055) = .90, *p* = .344 |
| Social Distress in Potential Romantic Relationship | *F*(1, 2055) = 2.40, *p* = .122 | M > F  *F*(1, 2055) = 4.10, *p* = .043,  *d* = .09 | *F*(1, 2055) = .45, *p* = .504 |
| Social Distress in Mixed-gender Groups | Single-sex > Coeducational  *F*(1, 2055) = 7.36, *p* = .007,  *d* = .12 | *F*(1, 2055) = .95, *p* = .330 | *F*(1, 2055) = 2.54, *p* = .111 |
| **Gender salience** | Single-sex > Coeducational  *F*(1, 2055) = 34.00, *p* < .001,  *d* = .26 | F > M  *F*(1, 2055) = 15.07, *p* < .001,  *d* = .17 | *F*(1, 2055) = .23, *p* = .633 |
| **Percentage of other-gender close friends** | Coeducational > Single-sex  *F*(1, 2055) = 36.73, *p* < .001,  *d* = .27 | M > F  *F* (1, 2055) = 4.86, *p* = .028,  *d* = .10 | *F*(1, 2055) = .00, *p* = .993 |
| **Percentage of other-gender friends** | Coeducational > Single-sex  *F*(1, 2055) = 158.01, *p* < .001,  *d* = .56 | *F*(1, 2055) = 3.13, *p* = .077 | *F*(1, 2055) = 10.75, *p* = .001 |

**Table C.** Results for alternative mediation models (Study 1: High school sample) – Mixed-gender friendships as outcome variable (Y).

|  | Model 1: X = School type (CE = 0, SS = 1); M_1_ = Gender salience, M_2_ = Mixed-gender anxiety; Y = Percentage of other-gender friends | | | | Model 2: X = School type (CE = 0, SS = 1); M_1_ = Gender salience, M_2_ = Mixed-gender anxiety; Y = Percentage of other-gender close friends | | | |
| --- | --- | --- | --- | --- | --- | --- | --- | --- |
|  | Total effect  (*p*) | Direct effect (c’)  (*p*) | Indirect effect | | Total effect  (*p*) | Direct effect (c’)  (*p*) | Indirect effect | |
|  |  |  | M_1_ (a_1_b_1_)  [LLCI, ULCI] | M_2_ (a_2_b_2_)  [LLCI, ULCI] |  |  | M_1_ (a_1_b_1_)  [LLCI, ULCI] | M_2_ (a_2_b_2_)  [LLCI, ULCI] |
| **M_2_** |  |  |  |  |  |  |  |  |
| Total mixed-gender anxiety | -.5502  (< .001)* | -.5244  (< .001)* | .0005  [-.0114, .0128] | -.0263  [-.0457, -.0101]* | -.2991  (< .001)* | -.2835  (< .001)* | .0001  [-.0128, .0134] | -.0156  [-.0302, -.0056]* |
| Fear of Negative Evaluation | -.5502  (< .001)* | -.5395  (< .001)* | .0005  [-.0120, .0128] | -.0112  [-.0245, -.0021]* | -.2991  (< .001)* | -.2943  (< .001)* | -.0001  [-.0131, .0137] | -.0047  [-.0147, -.0001]* |
| Social Distress in Potential Romantic Relationship | -.5502  (< .001)* | -.5316  (< .001)* | .0013  [-.0103, .0137] | -.0199  [-.0387, -.0042]* | -.2991  (< .001)* | -.2864  (< .001)* | .0007  [-.0119, .0141] | -.0134  [-.0271, -.0025]* |
| Social Distress in Mixed-gender Groups | -.5502  (< .001)* | -.5188  (< .001)* | -.0015  [-.0139, .0104] | -.0299  [-.0508, -.0130]* | -.2991  (< .001)* | -.2805  (< .001)* | -.0011  [-.0139, .0123] | -.0175  [-.0338, -.0072]* |

*Note*: In mediation models, the total effect refers to the association between the dependent variable (Y) and the independent variable (X), in which the indirect effect indicates the association mediated by the mediator (M) and the direct effect indicates the remaining association when the mediator is taken away. Coefficients were calculated with standardized values of gender salience, mixed-gender anxiety and percentage of other-gender friends and close friends. School type was not standardized because it is a dichotomous variable. * denotes statistical significance.

**Table D.** List of dating experience items from the Dating History Questionnaire [61].

| Dating Experience Items |
| --- |
| 1. Became romantically interested in men / women |
| 1. Had a “crush” on someone |
| 1. Felt at the time that you were “in love” with someone you were dating |
| 1. Dated someone, but with a group of friends |
| 1. Dated someone, just the two of you |
| 1. Dated a few different people over the year |
| 1. Dated one person on a fairly regular basis for at least one month |
| 1. Had a boyfriend / girlfriend |
| 1. Had a serious relationship |
| 1. Had a committed relationship in which you were planning to get engaged, married, or live together |
| 1. Got engaged, married, or lived with someone |

**Table E.** Correlations of study variables (Study 2: College sample).

|  | 1 | 2 | 3 | 4 | 5 | 6 | 7 | 8 | 9 | 10 | 11 | 12 | 13 | 14 | 15 | 16 | 17 | 18 | 19 | 20 |
| --- | --- | --- | --- | --- | --- | --- | --- | --- | --- | --- | --- | --- | --- | --- | --- | --- | --- | --- | --- | --- |
| 1. Student gender ^a^ |  | .013 | .004 | -.140^**^ | -.037 | -.054 | -.027 | .003 | -.005 | -.053 | .241^**^ | .068 | .037 | .036 | -.082 | .058 | .001 | -.010 | -.050 | -.031 |
| 1. School type ^b^ |  |  | -.040 | -.234^**^ | .060 | .029 | .086 | .062 | .121^**^ | -.048 | -.029 | .159^**^ | -.062 | -.067 | -.186^**^ | -.018 | -.036 | -.045 | -.005 | .021 |
| 1. Gender salience |  |  |  | .019 | -.008 | -.022 | -.007 | .027 | -.052 | -.088 | .020 | -.065 | -.057 | .124^**^ | .062 | .009 | .028 | .019 | .093^*^ | -.017 |
| 1. Percentage of other-gender close friends |  |  |  |  | -.186^**^ | -.122^**^ | -.234^**^ | -.161^**^ | -.023 | .127^**^ | -.066 | -.017 | .028 | -.028 | .016 | -.078 | .014 | .054 | -.007 | -.078 |
| 1. Anxiety in mixed-gender situations - Total |  |  |  |  |  | .931^**^ | .911^**^ | .804^**^ | .014 | -.149^**^ | -.015 | -.001 | .051 | -.024 | .064 | .729^**^ | -.048 | -.081 | -.064 | -.057 |
| 1. Anxiety in mixed-gender situations - Fear of Negative Evaluation |  |  |  |  |  |  | .739^**^ | .619^**^ | .019 | -.137^**^ | .001 | -.010 | .028 | -.052 | .057 | .665^**^ | -.034 | -.081 | -.076 | -.062 |
| 1. Anxiety in mixed-gender situations - Social Distress in Dating |  |  |  |  |  |  |  | .705^**^ | .009 | -.157^**^ | -.033 | .024 | .032 | .001 | .032 | .640^**^ | -.026 | -.054 | -.020 | -.027 |
| 1. Anxiety in mixed-gender situations - Social Distress in Mixed-gender Groups |  |  |  |  |  |  |  |  | .004 | -.089 | -.014 | -.021 | .110^*^ | .011 | .100^*^ | .659^**^ | -.094^*^ | -.084 | -.077 | -.065 |
| 1. Current monthly parental income |  |  |  |  |  |  |  |  |  | -.034 | .030 | .419^**^ | -.060 | -.078 | -.107^*^ | .042 | .069 | .056 | -.024 | .037 |
| 1. Student age |  |  |  |  |  |  |  |  |  |  | -.163^**^ | -.146^**^ | .049 | -.019 | .055 | -.100^*^ | .008 | .016 | -.028 | -.021 |
| 1. Faculty ^c^ |  |  |  |  |  |  |  |  |  |  |  | .066 | .062 | .008 | -.056 | .027 | -.006 | -.005 | -.025 | -.043 |
| 1. Parents’ education ^d^ |  |  |  |  |  |  |  |  |  |  |  |  | -.072 | -.179^**^ | -.190^**^ | .029 | -.017 | -.069 | -.105^*^ | -.015 |
| 1. Number of brothers |  |  |  |  |  |  |  |  |  |  |  |  |  | -.302^**^ | .038 | -.004 | -.032 | -.014 | -.018 | .001 |
| 1. Number of sisters |  |  |  |  |  |  |  |  |  |  |  |  |  |  | .046 | -.004 | -.054 | -.010 | .018 | -.017 |
| 1. Average school banding for all high schools attended |  |  |  |  |  |  |  |  |  |  |  |  |  |  |  | .052 | -.061 | -.094^*^ | -.019 | -.055 |
| 1. Social anxiety |  |  |  |  |  |  |  |  |  |  |  |  |  |  |  |  | -.135^**^ | -.146^**^ | -.142^**^ | -.121^**^ |
| 1. Sexual Attraction ^e^ |  |  |  |  |  |  |  |  |  |  |  |  |  |  |  |  |  | .676^**^ | .564^**^ | .575^**^ |
| 1. Sexual Behavior ^e^ |  |  |  |  |  |  |  |  |  |  |  |  |  |  |  |  |  |  | .676^**^ | .606^**^ |
| 1. Sexual Fantasy ^e^ |  |  |  |  |  |  |  |  |  |  |  |  |  |  |  |  |  |  |  | .597^**^ |
| 1. Sexual Identity ^e^ |  |  |  |  |  |  |  |  |  |  |  |  |  |  |  |  |  |  |  |  |

*Note*: ^a^ 1 = male, 2 = female. ^b^ 0 = coeducational, 1 = single-sex. ^c^ 1 = male-dominated, 2 = gender-balanced, 3 = female-dominated. ^d^ 1 = no schooling/pre-primary, 2 = primary, 3 = junior secondary, 4 = senior secondary, 5 = post-secondary, 6 = postgraduate. ^e^ 0 = other sex only, 1 = other sex mostly, 2 = other sex somewhat more, 3 = both sex equally, 4 = same sex somewhat more, 5 = same sex mostly, 6 = same sex only. * Correlation is significant at the .05 level (2-tailed). ** Correlation is significant at the .01 level (2-tailed).

**Table F.** 2 × 2 (School type × Student gender) ANOVA results (i.e., without covariates) of Study 2 (College sample).

| Dependent variables | Main effects | | Interactions |
| --- | --- | --- | --- |
|  | School type | Student gender | School type × Student gender |
| **Mixed-gender anxiety** |  |  |  |
| Total | *F*(1, 452) = 1.51, *p* = .220 | *F*(1, 452) = .48, *p* = .490 | *F*(1, 452) = 1.72, *p* = .190 |
| Fear of Negative Evaluation | *F*(1, 452) = .36, *p* = .548 | *F*(1, 452) = 1.22, *p* = .270 | *F*(1, 452) = .17, *p* = .678 |
| Social Distress in Dating | *F*(1, 452) = 3.10, *p* = .079 | *F*(1, 452) = .21, *p* = .647 | *F*(1, 452) = 2.51, *p* = .114 |
| Social Distress in Mixed-gender Groups | *F*(1, 452) = 1.46, *p* = .227 | *F*(1, 452) = .06, *p* = .801 | *F*(1, 452) = 5.37, *p* = .021 |
| **Gender salience** | *F*(1, 452) = .80, *p* = .373. | *F*(1, 452) = .03, *p* = .862 | *F*(1, 452) = .55, *p* = .460 |
| **Percentage of other-gender close friends** | Coeducational > Single-sex *F*(1, 452) = 26.08, *p* < .001,  *d* = .48 | M > F  *F*(1, 452) = 9.31, *p* = .002,  *d* = .29 | *F*(1, 452) = .24, *p* = .625 |

**Table G.** Results for “School type (single-sex vs. coeducational) × Student gender (male vs. female) × College year (first year vs. non-first year)” ANCOVAs in Study 2 (College sample).

| Dependent variables | Main effects | | | Interactions | | | |
| --- | --- | --- | --- | --- | --- | --- | --- |
|  | School type | Student gender | College year ^a^ | School type × Student gender | School type × College year | Student gender × College year | School type × Student gender × College year |
| **Mixed-gender anxiety** |  |  |  |  |  |  |  |
| Total | *F*(1, 437) = 6.80,  *p* = .009 | *F*(1, 437) = 4.79,  *p* = .029 | *F*(1, 437) = 2.10,  *p* = .148 | *F*(1, 437) = .02,  *p* = .895 | *F*(1, 437) = .69,  *p* = .407 | *F*(1, 437) = .19,  *p* = .665 | *F*(1, 437) = 1.60,  *p* = .207 |
| Fear of Negative Evaluation | *F*(1, 437) = 2.06,  *p* = .152 | *F*(1, 437) = 5.82,  *p* = .016 | *F*(1, 437) = .84,  *p* = .361 | *F*(1, 437) = .77,  *p* = .380 | *F*(1, 437) = 1.11,  *p* = .293 | *F*(1, 437) = .34,  *p* = .561 | *F*(1, 437) = 1.69,  *p* = .194 |
| Social Distress in Dating | *F*(1, 437) = 6.61,  *p* = .010 | *F*(1, 437) = 2.00,  *p* = .158 | *F*(1, 437) = 2.37,  *p* = .125 | *F*(1, 437) = .60,  *p* = .440 | *F*(1, 437) = 1.30,  *p* = .255 | *F*(1, 437) = .04,  *p* = .848 | *F*(1, 437) = .62,  *p* = .432 |
| Social Distress in Mixed-gender Groups | *F*(1, 437) = 8.86,  *p* = .003 | *F*(1, 437) = .84,  *p* = .359 | *F*(1, 437) = 1.41,  *p* = .236 | *F*(1, 437) = 1.67,  *p* = .197 | *F*(1, 437) = 1.02,  *p* = .313 | *F*(1, 437) = .01  *p* = .930 | *F*(1, 437) = .64,  *p* = .425 |
| **Gender salience** | *F*(1, 438) = .26,  *p* = .610 | *F*(1, 438) = .07,  *p* = .796 | *F*(1, 438) = 1.11,  *p* = .293 | *F*(1, 438) = .49,  *p* = .483 | *F*(1, 438) = .25,  *p* = .615 | *F*(1, 438) = .80,  *p* = .372 | *F*(1, 438) = .81,  *p* = .368 |
| **Percentage of other-gender close friends** | *F*(1, 438) = 20.81,  *p* < .001 | *F*(1, 438) = 8.27,  *p* = .004 | *F*(1, 438) = .99,  *p* = .321 | *F*(1, 438) = .20,  *p* = .657 | *F*(1, 438) = .00,  *p* = .989 | *F*(1, 438) = 1.31,  *p* = .254 | *F*(1, 438) = .73,  *p* = .394 |

Note: ^a^ Number of participants in each college year: 184 (Year 1), 124 (Year 2), 69 (Year 3), 66 (Year 4), 13 (Year 5). The college year variable was coded as 1 = first year, 2 = non-first year.

**Table H.** Results for alternative mediation model (Study 2: College sample) – Percentage of other-gender close friends as outcome variable (Y).

|  | Model: X = School type (CE = 1 SS = 2); M_1_ = Gender salience, M_2_ = Mixed-gender anxiety; Y = Percentage of other-gender close friends | | | |
| --- | --- | --- | --- | --- |
|  | Total effect  (*p*) | Direct effect (c’)  (*p*) | Indirect effect | |
|  |  |  | M_1_ (a_1_b_1_)  [LLCI, ULCI] | M_2_ (a_2_b_2_)  [LLCI, ULCI] |
| **M_2_** |  |  |  |  |
| Total mixed-gender anxiety | -.4775  (< .001)* | -.4364  (< .001)* | -.0008  [-.0193, .0049] | -.0404  [-.0892, -.0105]* |
| Fear of Negative Evaluation | -.4775  (< .001)* | -.4668  (< .001)* | -.0009  [-.0195, .0050] | -.0098  [-.0449, .0029] |
| Social Distress in Dating | -.4775  (< .001)* | -.4210  (< .001)* | -.0007  [-.0181, .0058] | -.0558  [-.1117, -.0167]* |
| Social Distress in Mixed-gender Groups | -.4775  (< .001)* | -.4420  (< .001)* | -.0012  [-.0212, .0047] | -.0344  [-.0812, -.0076]* |

*Note*: In mediation models, the total effect refers to the association between the dependent variable (Y) and the independent variable (X), in which the indirect effect indicates the association mediated by the mediator (M) and the direct effect indicates the remaining association when the mediator is taken away. Coefficients were calculated with standardized values of gender salience, mixed-gender anxiety and percentage of other-gender close friends. School type was not standardized because it is a dichotomous variable. * denotes statistical significance.

**Fig A.** Generic alternative mediation model.

In the alternative mediation model, a_1_b_1_ denotes the indirect effect of gender salience, a_2_b_2_ denotes the indirect effect of mixed-gender anxiety, and c’ denotes the direct effect of school type on mixed-gender friendships.


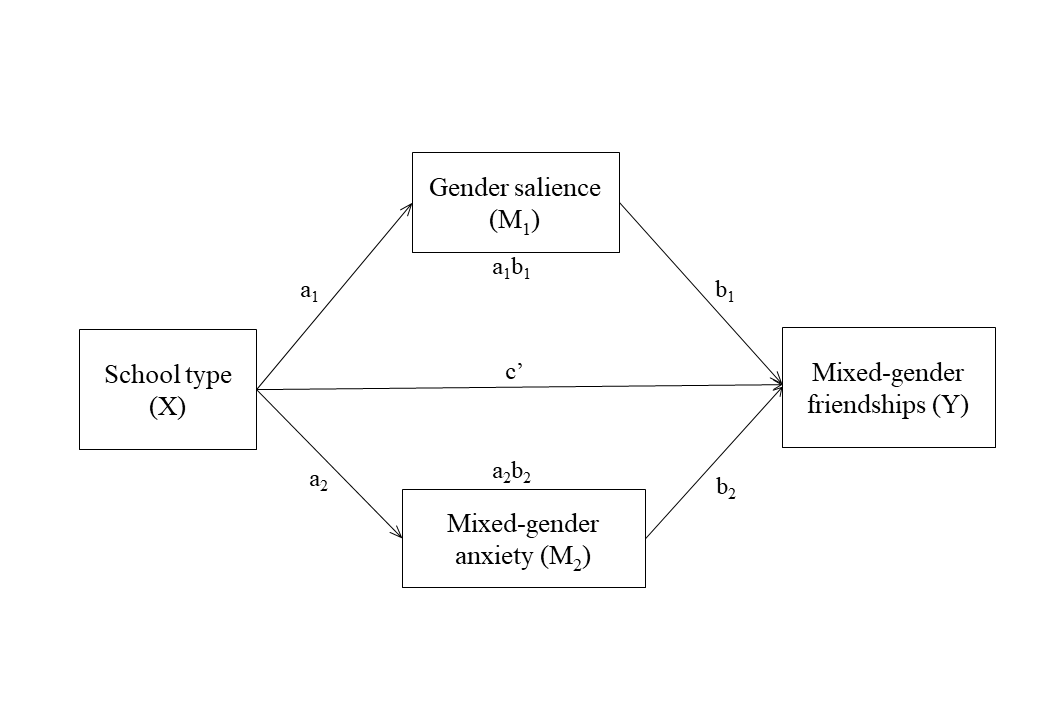

Supplement: S1 File — (DOC) [file pone.0208707.s001.doc]
